# Healthy ageing and oral health: priority, policy and public health

**DOI:** 10.1038/s41405-024-00262-z

**Published:** 2024-10-09

**Authors:** Rakhee Patel, Jennifer E. Gallagher

**Affiliations:** King’s College London, Faculty of Dentistry, Oral & Craniofacial Sciences, Dental Public Health, Centre for Host Microbiome Interactions, Denmark Hill Campus, Bessemer Road, SE5 9RS London, UK

**Keywords:** Dental epidemiology, Gerodontics

## Abstract

The global population ageing, and the pace of ageing is accelerating. Although people are living longer, these additional years are not being gained in health, and disability, chronic and long-term conditions increase with age. In response to the challenges of an ageing population, the United Nations and World Health Organisation declared 2021–2030 the Decade of Healthy Ageing, with the purpose of collaborative action to foster longer and healthier lives. This review explores the WHO public health framework for healthy ageing and global trends and policies, using the UK as an example of policy implementation. In response to the urgent need to consider the impact of ageing on oral health and oral healthcare systems, an integrated model for healthy ageing and oral health is proposed.

## Background

The global population is ageing, and the pace of population ageing is accelerating. According to the World Health Organisation (WHO) 2022 Health and Ageing Report, 1 in 6 people in the world will be aged 60 years or over by 2030; this represents a predicted increase from 1 billion in 2020 to 1.4 billion [1]. Looking further ahead, by 2050 the United Nations (UN) predicts the global population of people aged 60 years and older to double to 2.1 billion, and the number of people aged 80 years or older triple to 426 million [2].

There are challenges in how an ‘older person’ is defined. First, ageing is a complex process associated with biological, social and psychological changes, often presenting in an unsynchronised order, and significant heterogeneity in the needs of an ageing the population. Second, is chronological age; although the WHO [1], and UN [2], consider an older person to be over the age of 60 years, in many countries, context specific thresholds have been adopted, and ageing can only be vaguely linked to persons age in years. In the United Kingdom, for example, an older person has traditionally been defined as someone aged 65 years and over as this was the state retirement age, but with increasing life expectancy and changes to the retirement age, definitions are being reconsidered [3]. This includes consideration of ‘young old’ (65–74 years), ‘middle old’ (75–84 years) and ‘oldest old’ people ( ≥ 85 years) [4, 5].

Undoubtedly, population ageing can be viewed as a marker of improving health and successfully living longer. Starting in high income countries, such as Japan, where one third of the population is already aged over 60 years, now, every country in the world is experiencing growth in both the size and the proportion of older persons in the population [2].

**Fig. 1:**
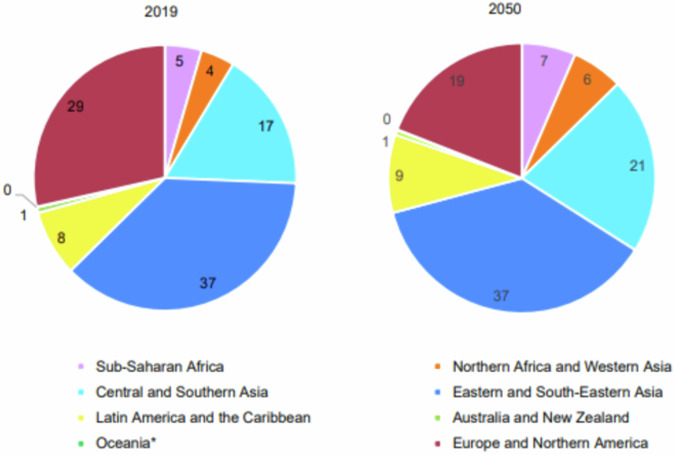
The distribution of percentage population aged 65 years and over by UN region from 2019 to 2050. Reproduced from *World Population Ageing 2019*, by United Nations Department of Economic and Social Affairs, ©(2020) United Nations. Reprinted with the permission of the United Nations.

As well the number and proportion of older persons within each region of the world increasing, there will be a shift in the global distribution of the ageing population (Fig. 1). Based on 2019 data, Eastern and South-Eastern Asia at 37% are home to the largest proportion of the world’s older population and will remain so in 2050. The second largest share of older persons live in Europe and Northern America (29%), which is expected to shrink to 19% by 2050. Central and Southern Asia host 17% of the global older population and is set to increase to 21% by 2050. Latin America and the Caribbean will see a small increase in its share of the world’s older population over this time period, from 8% in 2019 to 9% in 2050, as will Sub-Saharan Africa, Northern Africa and Western Asia from 5% to 7%, and 4% to 6%, respectively.

The potential support ratio is defined by the UN as the number of people of working age (25 to 64 years) per person aged 65 years or over [2]. It is predicted that population ageing will have a significant effect on the potential support ration with 48 countries (mostly in Europe, Northern America, Eastern Asia or South-Eastern Asia) having potential support ratios below two by 2050, i.e. there will be less than two working aged adults for every person aged 65 years and over. These low ratios highlight the impact of population ageing on both the labour market and economic performance of these countries, but also the financial pressures that they are likely to face in relation to health and social care, with those aged 85 years and over, the ‘oldest old’, having the highest care needs [6].

The increase in an ageing population has given rise to ‘ageism’, with common preconceptions of older people being a ‘burden’ [7]. However, there is huge diversity in ageing, with 80-year-olds who have the physical and mental capacities of a 30-year-olds, and younger people experiencing significant declines in capacities at an earlier stage. In their 2021 ‘Global Report of Ageism’, the WHO defines ageism as ‘the stereotypes (how we think), prejudice (how we feel) and discrimination (how we act) towards others or oneself based on age’ and reports that half the world’s population is ageist against older people, with a focus in Southeast Asia and Africa [7]. However, ageism is not limited to older people. In Europe, the only region for which data are available on all age groups, younger people reported more age discrimination than other age groups [7]. Furthermore, ageism also intersects and exacerbates other forms of disadvantage including those related to sex, race and disability.

The United Kingdom (UK) population is ageing, with the number of residents between 2011 and 2021 aged 65 years and over rising from 16.4% (9.2 million) in 2011, to 18.6% (over 11 million) in 2021 [8]. This included over half a million (527,900) people aged 90 years and over [6]. From predictions based on the last/previous Census [9], this is set to further increase to 1 in 4 of the UK population (20.4 million) aged over 65 years by 2066 [9]. The largest rise is predicted to be in people aged 85 years and over, which is set to double by 2041 to 4% of the population, and further treble by 2066 to 7% of the UK population [9]. Average life expectancy in the UK has also increased over the last 40 years, with current at birth estimates are 78.6 years for males and 82.6 years for females [10]. By the time a person reaches the age of 65 years, life expectancy for males is 18.3 years and 20.8 years for females [10].

Although more people are living for longer, they are not all living better. After the age of 65 years, UK disability-free life expectancy (DFLE) for men is 9.9 years and 9.8 years for women, which means men can expect to live 8.9 years, and women 11.3 years in poor health [11]. Additionally, over the last decade, disability free life expectancy (DFLE) has increased, but not at the same rate as life expectancy. Although men have gained 0.5 years of life expectancy, only 0.4 years have been disability free; for women, they have gained 0.2 years of life expectancy but only 0.1 years disability free [11]. So, we are living longer, but these extra years are not being gained in health, with a significant health and disability social gradient over the lifecourse [12].

Furthermore, the prevalence of almost all chronic and long-term conditions increases with age. The needs of a 65-year-old in many cases differ vastly from those of an 85-year-old because of physiological and psychological change. It is amongst the people aged 85-years and over that care needs are increased, and this is evidenced in the number of people requiring support with an ’activity of daily living’ (ADL) increasing by age. In the 2018 Health Survey for England, a third (32%) of 65–69-year-olds reported needing help with at least one ADL, rising to one in five (22%) for over 75-year-olds and 44% for age 80 and above [13].

The current state of our ageing population demonstrates a need to move towards healthy ageing rather than ageing as a disability.

## A decade of healthy ageing

Taking a global approach to the challenges of population ageing and associated health and care needs, the United Nations (UN) General Assembly declared 2021–2030 the UN Decade of Healthy Ageing. The World Health Organisation was tasked to lead the implementation of 10 years of collaborative action to foster longer and healthier lives [14–16], and has proposed collective action in four areas. First, changing how we think, feel and act towards age and ageism; second, developing communities in ways that foster the abilities of older people; third, delivering person-centred integrated care and primary health services responsive to older people; and fourth, providing older people with access to quality long-term care when required [14–16].

Healthy ageing is described as ‘the process of developing and maintaining the functional ability that enables well-being in older age’ [1]. This is based on the premise that the impact of ageing is not linear. Thus, rather than considering age in numerical terms, the WHO considers ageing in terms of people’s psychosocial function, whereby there are three categories of person:

1. A functionally independent older person can function independently although they have some chronic diseases.
2. A frail older person needs some assistance and may be living independently or in a care setting.
3. A functionally dependent older person who needs assistance and will be living in a care setting.

The WHO introduces two core components that influence and impact an individual’s ageing journey; these are their **intrinsic capacity** and their **environment** [1]. They suggest that the interplay of these two elements determines a person’s **functional ability** [1].

An individual’s intrinsic capacity is based on all the physical and mental capacities that a person can draw on. These may include a person’s mobility, hearing, vision, cognition, and psychological capacity. Building robust and resilient intrinsic capacity throughout one’s life is an essential part of healthy ageing. However, factors such as diseases or changes in psychological function can have profound effects and so a reserve of capacity is needed over the life course to ‘cope’ with age related changes.

The environment where people live inevitably shapes their lives to enable, or prohibit, an older person’s opportunity to undertake activities as they desire. This environment may be their home, community, or broader society, and all the factors within them, such as emotional support, attitudes, systems and services and technology, which can act as either barriers or facilitators [1].

When considered together, the environment and intrinsic capacity both determine a person’s ability to function to do what they value, i.e., their functional ability. This includes meeting their basic physical, social, and psychological needs, having purpose and contributing to society, building relationships, and having the opportunity to learn, grow and make decisions.

**Fig. 2:**
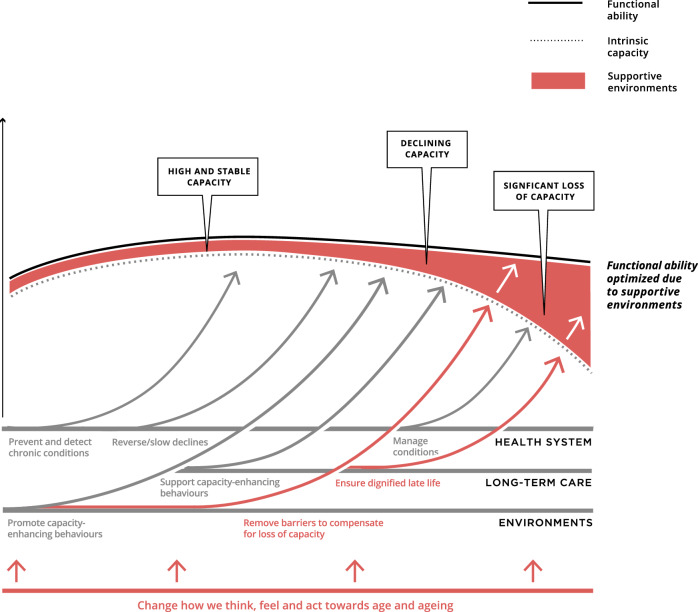
Trajectories of WHO healthy ageing to maximise functional ability. Reproduced from World Health Organisation, *Decade of healthy ageing: baseline report*, Copyright 2021.

**Fig. 3:**
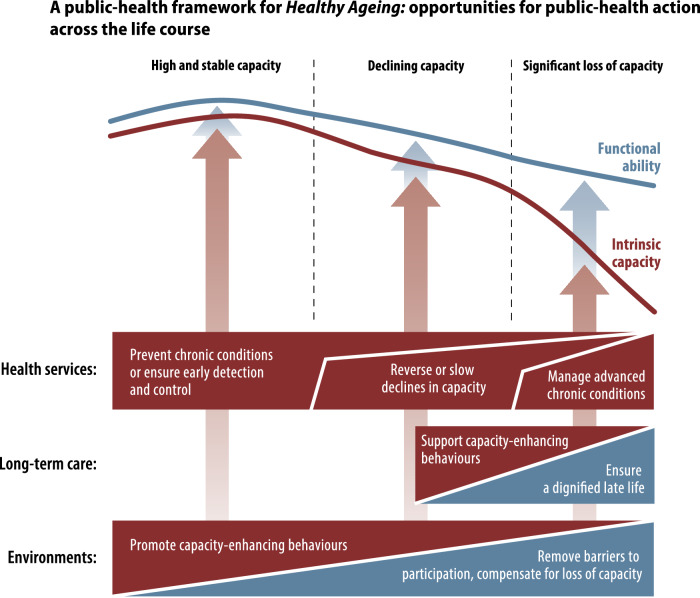
WHO (2015) Public Health Framework for Healthy Ageing: Opportunities for public-health action across the life course. Reproduced from World Health Organisation, *World report on Ageing and Health*, Copyright 2015.

Figure 2 demonstrates the interplay between health systems, longer term care and the environment to optimise personal functional ability through ageing:

Imperative to healthy ageing is appropriate prevention and intervention at earlier stages in the life course. Central to informing effective policy interventions to meet the challenges of a continually ageing population is a life course approach to identify early- and mid-life factors that have most impact on later lives. The WHO Public Health Framework for Healthy Ageing in 2015 [1] demonstrates the opportunities for public health action as functional ability and intrinsic capacity decline with age because of disease and the ageing process and how health services, long term care and the environment should be shaped to respond to declining capacity and ability. Supporting capacity enhancing behaviours and prevention of chronic diseases is vital throughout the ageing life course to facilitate a dignified late life where a person can be supported to end well. As people move through their ageing journey from having high and stable capacity, to declining and eventually loss of capacity towards end of life, it is important we consider when public health interventions are likely to be appropriate as well as effective. This is shown in the WHO diagram (Fig. 3).

## Oral health and ageing

Oral health deterioration in older age groups is also a global issue, with similar challenges of high levels of dental disease, challenging dental health systems and a need for clear policy. The Global Burden of Disease, Injury and Risk Factor Study (GBD), suggests that oral disorders affect more than 280 million older adults aged 70 years or more and is the 22^nd^ leading cause of global disability-adjusted life-years (DALYs) [17]. In a recent review, Kassebaum et al., estimated the global prevalence and incidence of oral conditions (untreated dental caries, periodontitis, edentulism, and other oral disorders) in older adults over 65 years to be 57% and 77% respectively [18]. There is significant variation in how countries are approaching these challenges, such as policy and improvement programmes, and there are considerable differences between low- and high-income country approaches [19]. The two most common oral diseases that pose a considerable public health burden in older people globally are dental caries and periodontal disease [20], although it should be noted that there are significant disparities in the epidemiologic indicators of oral disease used in the WHO regions collating the data in this study.

To address the high burden of untreated oral diseases and conditions worldwide a ‘global strategy on oral health’ was adopted by the 75th World Health Assembly [WHA] in May 2022, followed by a baseline report [14, 15], and Global Oral Health Action Plan [16]. This aligns with wider World Health Organisation [WHO] policies including Universal Health Coverage (UHC) [21]. The overall aspirations relate to facilitating UHC and to reduce the burden of oral disease [15]. Taking a public health approach, key priorities for action must be integrated with action during the decade of healthy ageing, thus, the upcoming decade will also be critical to for global oral health. This will involve developments in oral health promotion and disease prevention, health workforce developments, and consideration of access to essential oral health care, supported by governance, health informatics and research. There is great opportunity for innovation and collaboration in support the oral health of our ageing populations.

The primary oral diseases are periodontal disease and dental caries, although tooth wear, dry mouth and oral cancer are also relevant. Despite the connotations of losing teeth being a normal part of ageing, the process of ageing itself is not an independent risk factor for oral health. Rather, it is the factors associated with ageing, such as the prevalence of cardiovascular disease, diabetes, the impact of medication, changes in cognitive function, smoking and access to care that results in the risk of periodontal disease rising with age [22]; therefore, it is not surprising that periodontal disease risk increases with rising comorbidity and dependency [23–26].

In a recent global systematic review on coronal and root caries prevalence in older people, Chan et al., reported on the findings of 39 studies, 29 of which involved community dwelling older people, and 10 which reported the caries status of care home residents [27]. Challengingly, studies utilised different caries criteria, and there were differences in what was reported, including root caries, untreated caries, and some papers reporting either, others both. The prevalence of untreated caries ranged from 25% to 99% in community dwelling older people (Australia and South Africa respectively) and from 47% to 99% in institutionalised older adults (India and Vietnam respectively) [27]. The global median of mean prevalence of caries was 49% [27]. Interestingly, eleven studies focussed on the prevalence of untreated root caries with half of them conducted in residential home and/or day care centres. The prevalence of untreated root caries ranged from 8% to 74% in community dwelling older people (Finland and Brazil respectively), and from 30% to 96% in institutionalised older adults (Hong Kong and Vietnam respectively) [27]. The global median of mean prevalence of root caries was 46% [27]. These data suggest that dental caries in older people poses a significant burden and is worse in institutionalised elders for both untreated and root caries. This picture of poor oral cleanliness and high levels of disease is echoed throughout the national and international literature [28–30]. A particular challenge in older people is the incidence of root caries. In their recent prospective cohort study, Tokumoto et al., looked at the incidence of new and progression of existing root caries lesions in nursing home residents in Japan. They found that one in five participants had existing root caries at the start of their study, and by the one-year follow-up, almost 60% of participants had developed one new root caries lesion at tooth-level [31].

Oral health related quality of life across all indices is reported to be poor in older people, as demonstrated in the systematic review of 48 articles conducted by Azami-Aghdash et al., in 2021 [32]. This adds to the findings of secondary analysis of the UK Adult Dental Health Survey in 2017, whereby a subset of elderly participants data were used to assess their clinical normative need and reported OHIP-14 scores [33], the finding of which showed that the presence of active caries and of one or more of the pain, ulceration, fistula, abscess (PUFA) indicators were associated with impaired oral health related quality of life in older adults. Of equal importance, is the qualitative work undertaken by McGrath and Bedi over two decades ago [34], which employed a qualitative methodology to investigate the positive and negative impacts of poor oral health on quality of life of older people. Research in this area has found that older perceived oral health as having both positive and negative impacts on their quality of life, with the most common impacts being on eating and comfort [34, 35].

Fundamental to the prevention of dental diseases involves the regular effective removal of dental plaque from teeth and gum surfaces and fluoride interventions together with avoiding tobacco, enjoying a healthy diet; and, if drinking, advocate doing so within safer limits [36]. With ageing, dependency often increases, and people often become less compliant to oral care, and reliant on others for their personal care, including oral care which presents challenges. Furthermore, dietary changes, comorbidity and polypharmacy result in increased risk of dental diseases as outlined below.

## Long term conditions and oral health

General health needs and oral health are intrinsically linked acting synergistically through the oral manifestations of certain systemic diseases. The most prevalent chronic conditions often share the same ‘common risk factors’ [37] with oral health conditions. In many conditions, oral health and general health exist in a bi-directional relationship with general health conditions impacting oral health. However, historically the oral cavity has been separated from the rest of the body, and thus research on the relationship between oral health and general health has been relatively recent and is still emerging.

### Respiratory impacts

In their 2006 systematic review, Azarpazhooh and Leake found fair evidence of an association between pneumonia and oral health [38]. Further to this, Manger et al., [39] confirmed this in their rapid review of the relationship between oral health and pulmonary disease and found strong evidence that frail populations would have lower incidence of pneumonia with regular oral hygiene interventions [39], with significant impacts on oral health related quality of life [40].

### Diabetes

Diabetes and oral health have been an area of much interest over the last decade, and there is some evidence of a bidirectional relationship between diabetes and oral health and the management of conditions, most notably for periodontitis on a patient’s diabetes [25]. Preshaw et al. [26] reported in their review, that the risk for periodontitis increases two to three times in people with diabetes, irrespective of type, compared to individuals without, and the level of glycaemic control being key in determining risk.

### Cardiovascular disease

There is evidence linking an association between several oral conditions, including periodontitis, caries and tooth loss and atherosclerotic cardiovascular disease [25, 41], although not a causative relationship [42]. It should be noted that this association is limited to atherosclerotic diseases, that is, coronary heart disease, stroke, and peripheral vascular disease [43]. There is little evidence to associate oral health conditions and non-atherosclerotic disorders, such as hypertension, arrythmias or heart failure (25).

### Cognitive ability

Several studies have shown an association between cognitive ability and oral health [44–46] across the life course. It is not surprising that as cognitive function declines so does oral health, as oral health behaviours change. Infrequent tooth brushing and changes in eating habits leads to increased plaque accumulation and increased risk of dental caries. Naorungoaj et al., [47] considered change in oral health as part of their six-year cohort study of middle-aged adults and found that over this period of time, a decline in cognitive function was associated with less frequent toothbrushing, higher plaque levels and greater odds of edentulism [47, 48]. Several recent reviews have shown that older people with dementia have worse oral health, with more retained roots, increased prevalence of coronal and root caries and reduced access to dental services, when compared to older people without dementia [49–52]. The most consistent association is between dementia and poor oral hygiene.

### Nutrition

It has long been reported that there is a complex relationship between oral health status, food choice, nutritional status, and general health status [53–56]. The relationship between nutrition and oral health is co-dependent, whereby the condition of a person’s oral health status will impact their food choices, and their food choices will affect their risk of dental disease. A common example of this is in the prescribing of high calorie food drink supplements for people who are losing weight. As well as being a high calorie food supplement, many of these drinks are high in sugar and as they are advised to drink small quantities throughout the day, the patient’s risk for dental caries rises. What is often not considered, is the possibility that the oral condition is contributing to the persons inability to eat and ensuing weight loss. Rauen et al., [57] found a statistically significant association between a highly compromised dentition and thinness of elderly residents in care homes in Brazil. In contrast, those with a less compromised dentition had better nutrition and were classified as being overweight. This relationship between tooth number, oral function, and body mass index (BMI) in people over 65 years is also shown in Sheiham et al.’s, analysis of the 1998 National Diet and Nutrition Survey [58]. Dion et al., (2007) undertook statistical analysis of a large sample of elderly care residents correcting for other risk factors for malnutrition and found that for every 10% reduction in masticatory function (equivalent to the loss of a pair of molar teeth), the risk of malnutrition increases significantly and continuously by 1.15 times [59].

## Case study: oral health throughout the ageing life course in the UK

Epidemiological data in the UK show that as well as the number and proportion of older people increasing, the number of people retaining some of their own teeth is rising. The 2009 Adult Dental Health Survey reported 53% of adults aged 85 years and over had retained some of their dentition, with an average of 14 teeth present [60]. The 2021 online survey suggests that amongst adults in England aged 75 years and over, just over half (52%) had 21 or more natural teeth which is consistent with a functional dentition [61]. Over recent years, there has been a shift from dental extractions and the provision of dentures to patients wanting to retain their dentition for longer by undertaking complex and costly restorative treatment. This means we now have a cohort of older people who can benefit from prevention to maintain their oral health. In the 2015 review of oral health surveys of older people, Public Health England reported that older adults living in residential and nursing care homes are more likely to be edentulous, and less likely to have a functional dentition [61] In the 2021 Oral Health Survey for England, denture-wearing was highest amongst adults aged 75 years and over (39%) [62].

**Fig. 4:**
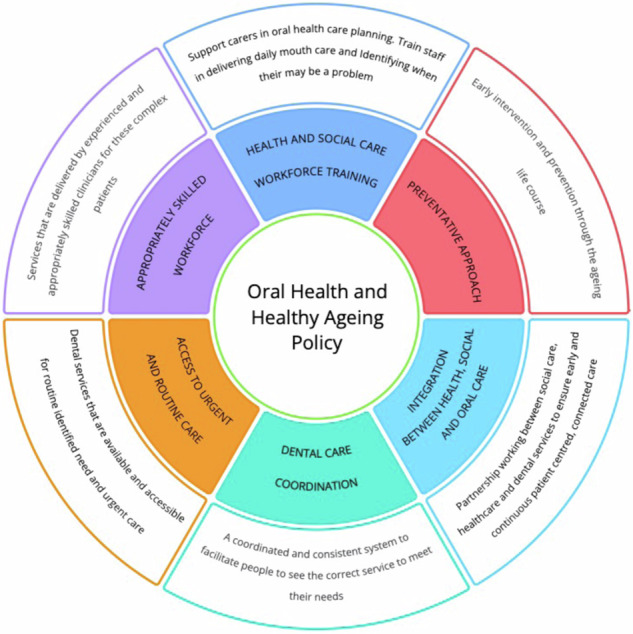
An integrated model for healthy ageing and oral health developed from the findings of the FIouride Interverventions in Care Homes Trial (FInCH).

The global movement towards a decade of healthy ageing, and oral health policies make this an opportune and important time to shape healthcare for our increasingly ageing population. A broader lens with a focus on primary prevention of chronic diseases including oral diseases, a common risk factor approach and consideration of the overall food environment is needed. To realise significant change, upstream, midstream, and downstream actions should be advocated for including the wider social and commercial determinants of health and their impact on healthy ageing and oral health. To have long term impact on the oral health of our ageing population, action is needed at several levels as shown in Fig. 4:

### Health and social care workforce training

In the UK, policy drivers and guidelines advocate for adoption of oral care policies, training of the social care workforce and access to dental care. This has been focused on care homes/institutionalised care setting for older persons and fail to take a life course approach to ageing more broadly. The 2016 National Institute for Clinical Excellence (NICE) NG48 guidelines relating to oral health in care homes [63] advise the need for a care home oral health policy, oral health assessments and mouthcare plans for all care home residents on entry, training of care staff to ensure knowledge and skills were updated and suitable arrangement for access to dental services. However, in its original format, it was of limited use to care home teams, with care teams reporting it to not reflect the contextual reality of daily care within care homes [64].

In Scotland and Wales, national policy has driven the importance of oral health for older people. The Scottish ‘Action Plan to Improve and Modernise NHS Dentistry’ in 2005 [65], identified dependent older people as a priority group, and the 2012 Scottish Governments National Oral Health Improvement Plan included action to improve the oral health of older people in care homes. Part of this was the inception of the ‘Caring for Smiles’ national programme [66] which includes accredited training and support for the care home teams in line with NG48. A similar policy approach was taken in Wales, with the Welsh Government committing ring fenced funding for the ‘Gwên am byth’ national multi agency oral health improvement programme since 2015 [67].

This has not been the case in England. In 2019, the independent regulator of health and social care in England, the Care Quality Commission (CQC), released their review of oral health in care homes, ‘Smiling Matters’ originally published in 2019, which was updated in 2023 [68]. This review highlighted significant failures in the adoption of NG48 in care homes across England and recommended a cross sector approach including reinforcement of the guidance, thus making oral health training mandatory for care staff, oral health check-ups for all residents on entry and a multi-agency group to be formed to raise awareness. More significantly, they introduced its plan to include oral health in their care home inspections. Although this CQC review laid the foundation for England’s policy lining up with Scotland and Wales, no national policy was set, with even the recent ‘Faster, simpler and fairer: our plan to recover and reform NHS dentistry’ in 2024 [69], failing to mention our ageing population.

### Preventative approach

**Fig. 5:**
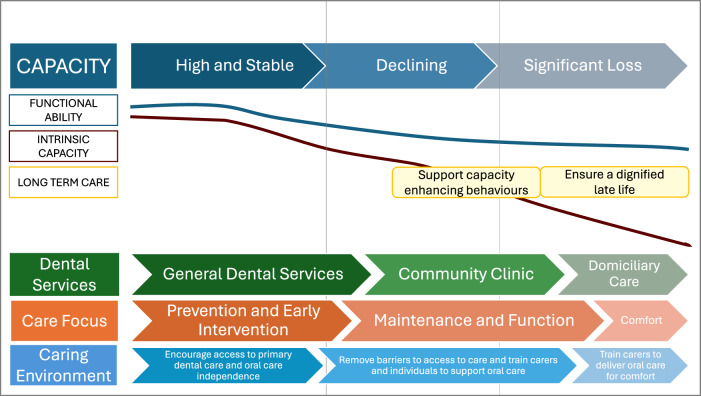
A dental public health approach to health ageing. Adapted from World Health Organisation, *World report on Ageing and Health*, Copyright 2015. WHO is not responsible for the content or accuracy of this adaptation.

From a public health perspective, the importance of early intervention and prevention is widely known, and advocacy for policies that support a preventative focus and pragmatic oral care through ageing is needed to ensure support policy change. Taking a public health approach by acting earlier in the life course, and further upstream and as a network with partners such as local public services is essential to healthy ageing and will support the health and social care system with the pressures of an ageing population. Figure 5 reimagines the dental public health approach in the context of the WHO Public Health Framework for Healthy Ageing.

Taking an evidence-based approach to prevention, be they through traditional methods of fluoride delivery through fluoride varnish and or high fluoride toothpaste for primary caries prevention, and the emerging use of silver diamine fluoride [70–72], in the arrest of existing caries, in this vulnerable group should be a core part of the public health response.

### Integration between health, social and oral care

The evidence linking oral health and general health conditions and consequent prescribing patterns demonstrates the importance of integrating oral health into wider healthcare anticipatory care approaches. Anticipatory care is in place to intervene before the persons condition deteriorates, and therefore takes a person-centred, proactive approach. In terms of oral health, this translates to generating an understanding of a person’s priorities, pragmatic treatment planning to ensure maintenance and function. Integration with health and social care colleagues from point of diagnosis of a general health condition with oral health implications is essential to ensure people are supported throughout and avoid the consequences that arise with a deteriorating dentition.

### Access to care and care coordination

The impacts of poor oral health mean visiting dental professionals routinely for preventative advice and care is imperative [36]. However, UK trend data shows that dental check-up attendance falls significantly after the ages of 64 years and continues to reduce through the ageing journey [73, 74]. Recall guidance set by the National Institute for Health Published research [75] advocates that patients should be ‘risk assessed’ based on clinical, medical, and psychosocial needs and based on this assessment, a recall attendance period be advised, ranging from 3 months (for those at high risk of dental disease) up to 2 years (for those at low risk). When considering this guidance against the high levels of dental need, coupled with polypharmacy and dependency, it can be considered that as people age, they are likely to be at higher risk of disease and should be on a shorter recall period, and yet there is little consistency across the UK in the offer of urgent or routine care for the vulnerable elderly [76]. One of the key issues is over the last two decades, there has been a marked reduction in the provision of domiciliary care which is likely to be increasingly required by our ageing dependent population [77]. There is significant local and national variation and the absence of a national specification in the approach to which services a person should be directed to, what standard of clinical care is expected, and which clinical setting is suitable, be it general dental services, community dental services or domiciliary care based. A standardised and coordinated approach to oral care delivery for our ageing population is vital.

There are also challenges from a patient’s perspective. The cost of dental care is a significant barrier for older adults [78], with variation in exemptions across the UK [76]. With limited access to funds in a care home, unknown costs can be an issue for many residents and their families both in the UK and abroad [76, 78, 79]. Patient perceptions of needs also differ, resulting in many elderly people only accessing care when there is a problem, with a ‘passive acceptance’ of oral health [79].

The challenges around access to dental care need to be balanced with the realities of undertaking clinical treatment for complex care home residents, be it in a domiciliary or clinical setting. This patient group presents with unique complexities such as medical complications, limited mobility and compliance issues; therefore, when a need for dental treatment is identified in many cases the complex the range of dental treatments deliverable is restricted. As a result of the complexities associated with delivering care, there are disparities in research and clinical reasoning as to whether disease presence equates to treatment need for care home residents. [80]. This highlights the need for a national clinical standard. Services should be codeveloped in partnership with social care experts, so that there is a shared understanding of the challenges from both perspectives and opportunities for novel and useful solutions are explored.

As well as limitations around the availability and accessibility of dental care, there are complexities around workforce. Treating older people with complex physiological, medical, and behavioural needs is challenging and requires additional skills. Currently, these come with training and exposure, which is not a core part of undergraduate or even postgraduate training widely. In the current dental climate, action will inevitably require innovation in how and where dental care is offered for our ageing dependent population, but also presents workforce opportunities within the dental team and wider. With the diverse skill mix model in the UK [81, 82], novel ways of utilising skill mix, and the roles of the dental team as well as opportunities for integrating the workforce across health, dental and social care should be considered to ensure an adequately skilled workforce to care for our vulnerable ageing population.

## Conclusion

Healthy ageing is an important global issue, and the impact of an ageing population on oral health and oral healthcare systems needs urgent consideration. The impact that the oral condition alone has on an individual’s quality of life and function is of vital importance, made further relevant by the bidirectional relationship with general health. It is imperative that measures to plan for services which are patient-centred, and prevention focused are put in place, both for the quality of life of this cohort of people as well as the implications for health systems. With the inter-relationship between general health and poor oral health, prevention of oral diseases and supporting access to routine and urgent care together with advocacy to support prioritisation of health needs within policy and practice is required to ensure the maintenance and function of the ageing dentition. Finally, there is a paucity of research on the social value of oral health of older population and impact of public health and social care policy interventions on healthy ageing including oral health which needs urgent attention.

## Data Availability

Not applicable.

## References

[1] Decade of healthy ageing: baseline report. Geneva: World Health Organization; (2020). Licence: CC BY-NC-SA 3.0 IGO. 9789240017900-eng (2).pdf.

[2] United Nations, Department of Economic and Social Affairs, Population Division (2019). World Population Prospects 2019: Highlights (ST/ESA/SER.A/423). https://population.un.org/wpp/Publications/Files/WPP2019_Highlights.pdf.

[3] Office for National Statistics. Living longer: is age 70 the new age 65? (2019). https://www.ons.gov.uk/peoplepopulationandcommunity/birthsdeathsandmarriages/ageing/articles/livinglongerisage70thenewage65/2019-11-19#implications-and-limitations.

[4] Baltes PB, Smith J. New Frontiers in the Future of Aging: From Successful Aging of the Young Old to the Dilemmas of the Fourth Age. Gerontology. 2003;49:123–35. 10.1159/000067946. 12574672 10.1159/000067946

[5] Cohen-Mansfield J, Shmotkin D, Blumstein Z, Shorek A, Eyal N, Hazan H. CALAS Team. The old, old-old, and the oldest old: continuation or distinct categories? An examination of the relationship between age and changes in health, function, and wellbeing. Int J Aging Hum Dev. 2013;77:37–57. 10.2190/AG.77.1.cPMID: 23986979. 23986979 10.2190/AG.77.1.c

[6] Abell, J, Neil, A-S, James, B, David, B, & Josaine, B (2018). The Dynamics of Ageing. Evidence from the English longitudinal study of ageing 2002–16. https://www.ucl.ac.uk/drupal/site_iehc/sites/iehc/files/elsawave8report.pdf.

[7] Global report on ageism. Geneva: World Health Organization; (2021). Licence: CC BY-NC-SA 3.0 IGO. https://www.who.int/publications/i/item/9789240016866.

[8] https://www.ons.gov.uk/peoplepopulationandcommunity/birthsdeathsandmarriages/ageing/articles/profileoftheolderpopulationlivinginenglandandwalesin2021andchangessince2011/2023-04-03 Office for National Statistics (ONS) released 3 April 2023, ONS website, article, Profile of the older population living in England and Wales in 2021 and changes since 2011.

[9] Office for National Statistics (ONS) released 13^th^ August 2018, ONS website, article, Living longer: how our population is changing and why it matters. https://www.ons.gov.uk/peoplepopulationandcommunity/birthsdeathsandmarriages/ageing/articles/livinglongerhowourpopulationischangingandwhyitmatters/2018-08-13.

[10] Office for National Statistics (ONS) released 12^th^ January 2022, ONS website, data tables, Past and projected period and cohort life tables: 2020-based, UK, 1981 to 2070. https://www.ons.gov.uk/peoplepopulationandcommunity/birthsdeathsandmarriages/lifeexpectancies.

[11] https://www.ons.gov.uk/peoplepopulationandcommunity/healthandsocialcare/healthandlifeexpectancies/datasets/healthstatelifeexpectancyatbirthandatage65bylocalareasuk Office for National Statistics (ONS) released 11^th^ December 2019, ONS website, data tables Health state life expectancy at birth and at age 65 years by local areas, UK.

[12] Centre for Ageing Better. The state of Ageing 2022 online report published March 2022. https://ageing-better.org.uk/health-state-ageing-2022.

[13] Health and Social Care Information Centre. Health Survey for England 2018: Social care for older adults published online by NHS Digital on 3^rd^ December 2019. http://healthsurvey.hscic.gov.uk/media/81673/HSE18-Social-Care-rep.pdf.

[14] World Health Organisation. Landmark global strategy on oral health adopted at World Health Assembly 75- article. May 2022. https://www.who.int/news-room/feature-stories/detail/landmark-global-strategy-on-oral-health-adopted-at-world-health-assembly-75.

[15] Global oral health status report: towards universal health coverage for oral health by 2030. Geneva: World Health Organization; 2022. Licence: CC BY-NC-SA 3.0 IGO. https://www.who.int/publications/i/item/9789240061484.

[16] World Health Organisation (January 2024) Draft Global Oral Health Action Plan (2023–2030). Available at: https://www.who.int/publications/m/item/draft-global-oral-health-action-plan-(2023-2030).

[17] Collaborators, G. D. A. I. Global burden of 369 diseases and injuries in 204 countries and territories, 1990-2019: a systematic analysis for the Global Burden of Disease Study 2019. Lancet. 2020;396:1204–22. 10.1016/S0140-6736(20)30925-9. 33069326 10.1016/S0140-6736(20)30925-9 PMC7567026

[18] Kassebaum NJ, Smith AGC, Bernabé E, Fleming TD, Reynolds AE, Vos T, et al. Global, Regional, and National Prevalence, Incidence, and Disability-Adjusted Life Years for Oral Conditions for 195 Countries, 1990-2015: A Systematic Analysis for the Global Burden of Diseases, Injuries, and Risk Factors. J Dent Res. 2017;96:380–7. 28792274 10.1177/0022034517693566 PMC5912207

[19] Jiang CM, Chu CH, Duangthip D, Ettinger RL, Hugo FN, Kettratad-Pruksapong M, et al. Global Perspectives of Oral Health Policies and Oral Healthcare Schemes for Older Adult Populations. Front Oral Health. 2021;2:703526. 10.3389/froh.2021.703526. 35048040 10.3389/froh.2021.703526 PMC8757822

[20] Petersen PE, Kandelman D, Arpin S, Ogawa H. Global oral health of older people-call for public health action. Community Dent Health. 2010;27:257–67. DecPMID: 21313969. 21313969

[21] World Health Organisation (2023): WHO Universal Health Coverage Factsheet. Available at: https://www.who.int/news-room/fact-sheets/detail/universal-health-coverage-(uhc).

[22] Graham L, Turner W. Periodontal Disease in an Ageing Population: Key Considerations in Diagnosis and Management for the Dental Healthcare Professional. Prim Dent J. 2020;9:23–28. 10.1177/2050168420943407. 32940593 10.1177/2050168420943407

[23] Naorungroj S, Schoenbach VJ, Wruck L, Mosley TH, Gottesman RF, Alonso A, et al. Tooth loss, periodontal disease, and cognitive decline in the Atherosclerosis Risk in Communities (ARIC) study. Community Dent Oral Epidemiol. 2015b;43:47–57. 10.1111/cdoe.12128. 25363061 10.1111/cdoe.12128 PMC4303516

[24] D’Aiuto F, Gable D, Syed Z, Allen Y, Wanyonyi KL, White S, et al. Evidence summary: The relationship between oral diseases and diabetes. Br Dent J. 2017;222:944–8. 10.1038/sj.bdj.2017.544. 28642531 10.1038/sj.bdj.2017.544

[25] Dietrich T, Webb I, Stenhouse L, Pattni A, Ready D, Wanyonyi KL, et al. Evidence summary: the relationship between oral and cardiovascular disease. Br Dent J. 2017;222:381–5. 28281612 10.1038/sj.bdj.2017.224

[26] Preshaw PM, Bissett SM. Periodontitis and diabetes. Br Dent J. 2019;227:577–84. 10.1038/s41415-019-0794-5. 31605062 10.1038/s41415-019-0794-5

[27] Chan, AKY, Tamrakar, M, Jiang, CM, Lo, ECM, Leung, KCM, & Chu, CH (2021). A Systematic Review on Caries Status of Older Adults. Int J Environ Res Public Health, 18. 10.3390/ijerph182010662. 10.3390/ijerph182010662 PMC8535396 34682414

[28] Patel R, Fitzgerald R, Warburton F, Robertson C, Pitts NB, Gallagher JE. Refocusing dental care: A risk-based preventative oral health programme for dentate older people in UK care homes. Gerodontology. 2022;39:131–8. 10.1111/ger.12543. 33586205 10.1111/ger.12543

[29] Karki AJ, Monaghan N, Morgan M. Oral health status of older people living in care homes in Wales. Br Dent J. 2015b;219:331–4. 10.1038/sj.bdj.2015.756. 26450249 10.1038/sj.bdj.2015.756

[30] Gluhak C, Arnetzl GV, Kirmeier R, Jakse N, Arnetzl G. Oral status among seniors in nine nursing homes in Styria, Austria. Gerodontology. 2010a;27:47–52. 10.1111/j.1741-2358.2009.00281.x. 19371391 10.1111/j.1741-2358.2009.00281.x

[31] Tokumoto K, Kimura-Ono A, Mino T, Osaka S, Numoto K, Koyama E, et al. Risk factors for root caries annual incidence and progression among older people requiring nursing care: A one-year prospective cohort study. J Prosthodont Res. 2022;66:250–7. 10.2186/jpr.JPR_D_20_00272. 34470983 10.2186/jpr.JPR_D_20_00272

[32] Azami-Aghdash S, Pournaghi-Azar F, Moosavi A, Mohseni M, Derakhshani N, Kalajahi RA. Oral Health and Related Quality of Life in Older People: A Systematic Review and Meta-Analysis. Iran J Public Health. 2021;50:689–700. 10.18502/ijph.v50i4.5993. 34183918 10.18502/ijph.v50i4.5993 PMC8219627

[33] Masood M, Newton T, Bakri NN, Khalid T, Masood Y. The relationship between oral health and oral health related quality of life among elderly people in United Kingdom. J Dent. 2017;56:78–83. 10.1016/j.jdent.2016.11.002. 27825838 10.1016/j.jdent.2016.11.002

[34] Mcgrath C, Bedi R. A study of the impact of oral health on the quality of life of older people in the UK-findings from a national survey. Gerodontology. 1998;15:93–98. 10.1111/j.1741-2358.1998.00093. 10530183 10.1111/j.1741-2358.1998.00093.x

[35] Atanda AJ, Livinski AA, London SD, Boroumand S, Weatherspoon D, Iafolla TJ, et al. Tooth retention, health, and quality of life in older adults: a scoping review. BMC Oral Health. 2022;22:185. 10.1186/s12903-022-02210-5. May 18PMID: 35585618; PMCID: PMC9118621. 35585618 10.1186/s12903-022-02210-5 PMC9118621

[36] Department of Health (2009). Delivering Better Oral Health: An evidence-based toolkit for prevention. Delivering better oral health: an evidence-based toolkit for prevention-GOV.UK. (www.gov.uk).

[37] Sheiham A, Watt RG. The common risk factor approach: a rational basis for promoting oral health. Community Dent Oral Epidemiol. 2000;28:399–406. 10.1034/j.1600-0528.2000.028006399.x. 11106011 10.1034/j.1600-0528.2000.028006399.x

[38] Azarpazhooh A, Leake JL. Systematic review of the association between respiratory diseases and oral health. J Periodontol. 2006;77:1465–82. 10.1902/jop.2006.060010. 16945022 10.1902/jop.2006.060010

[39] Manger D, Walshaw M, Fitzgerald R, et al. Evidence summary: the relationship between oral health and pulmonary disease. Br Dent J. 2017;222:527–33. 10.1038/sj.bdj.2017.315. 28387268 10.1038/sj.bdj.2017.315

[40] Li S, Ning W, Wang W, Ziebolz D, Acharya A, Schmalz G, et al. Oral Health-Related Quality of Life in Patients With Chronic Respiratory Diseases-Results of a Systematic Review. Front Med (Lausanne). 2022;8:757739. 10.3389/fmed.2021.757739. 35096862 10.3389/fmed.2021.757739 PMC8790480

[41] Kotronia E, Brown H, Papacosta AO, Lennon LT, Weyant RJ, Whincup PH, et al. Oral health and all-cause, cardiovascular disease, and respiratory mortality in older people in the UK and USA. Sci Rep. 2021;11:16452. 10.1038/s41598-021-95865-z. 34385519 10.1038/s41598-021-95865-z PMC8361186

[42] Febbraio M, Roy CB, Levin L. Is There a Causal Link Between Periodontitis and Cardiovascular Disease? A Concise Review of Recent Findings. Int Dent J. 2022;72:37–51. 10.1016/j.identj.2021.07.006. 34565546 10.1016/j.identj.2021.07.006 PMC9275186

[43] Gianos E, Jackson EA, Tejpal A, Aspry K, O’Keefe J, Aggarwal M, et al. Oral health and atherosclerotic cardiovascular disease: A review. Am J Prev Cardiol. 2021;7:100179. 10.1016/j.ajpc.2021.100179. 34611631 10.1016/j.ajpc.2021.100179 PMC8387275

[44] Wu B, Fillenbaum GG, Plassman BL, Guo L. Association Between Oral Health and Cognitive Status: A Systematic Review. J Am Geriatr Soc. 2016;64:739–51. 10.1111/jgs.14036. 27037761 10.1111/jgs.14036 PMC4986993

[45] Nangle MR, Riches J, Grainger SA, Manchery N, Sachdev PS, Henry JD. Oral Health and Cognitive Function in Older Adults: A Systematic Review. Gerontology. 2019;65:659–72. 10.1159/000496730. 30904915 10.1159/000496730

[46] Daly B, Thompsell A, Sharpling J, Rooney YM, Hillman L, Wanyonyi KL, et al. Evidence summary: the relationship between oral health and dementia. Br Dent J. 2018;223:846–53. 10.1038/sj.bdj.2017.992. 29192686 10.1038/sj.bdj.2017.992

[47] Naorungroj S, Schoenbach VJ, Beck J, Mosley TH, Gottesman RF, Alonso A, et al. Cross-Sectional associations of oral health measures with cognitive function in late middle-aged adults: a community-based study. J Am Dent Assoc. 2013;144:1362–71. 24282266 10.14219/jada.archive.2013.0072 PMC4955404

[48] Naorungroj S, Slade GD, Beck JD, Mosley TH, Gottesman RF, Alonso A, et al. Cognitive decline and oral health in middleaged adults in the ARIC study. J Dent Res. 2013a;92:795–801. 23872988 10.1177/0022034513497960 PMC3744272

[49] Manchery N, Henry JD, Lam BCP, et al. Memory decline in older individuals predicts an objective indicator of oral health: findings from the Sydney Memory and Ageing Study. BMC Oral Health. 2022;22:93. 10.1186/s12903-022-02128-y. 35346157 10.1186/s12903-022-02128-y PMC8962025

[50] Jockusch J, Hopfenmüller W, Nitschke I. Influence of cognitive impairment and dementia on oral health and the utilization of dental services : Findings of the Oral Health, Bite force and Dementia Study (OrBiD). BMC Oral Health. 2021;21:399. 10.1186/s12903-021-01753-3. 34391408 10.1186/s12903-021-01753-3 PMC8364098

[51] Scambler S, Curtis S, Manthorpe J, Samsi K, Rooney YM, Gallagher JE. The mouth and oral health in the field of dementia. Health. 2023;27:540–58. 10.1177/13634593211049891. 34727785 10.1177/13634593211049891 PMC10197156

[52] Kc S, Aulakh M, Curtis S, Scambler S, Gallagher JE. Perspectives of community-dwelling older adults with dementia and their carers regarding their oral health practices and care: rapid review. BDJ Open. 2021;7:36. 10.1038/s41405-021-00091-4. 34811365 10.1038/s41405-021-00091-4 PMC8608883

[53] Chan AKY, Tsang YC, Jiang CM, Leung KCM, Lo ECM, Chu CH. Diet, Nutrition, and Oral Health in Older Adults: A Review of the Literature. Dent J **2023**;11:222. 10.3390/dj11090222. 10.3390/dj11090222 PMC10528506 37754342

[54] Algra Y, Haverkort E, Kok W, Etten-Jamaludin FV, Schoot LV, Hollaar V, et al. The Association between Malnutrition and Oral Health in Older People: A Systematic Review. Nutrients. 2021;13:3584. 10.3390/nu13103584. 34684584 10.3390/nu13103584 PMC8541038

[55] Van Lancker A, Verhaeghe S, Van Hecke A, Vanderwee K, Goossens J, Beeckman D. The association between malnutrition and oral health status in elderly in long-term care facilities: a systematic review. Int J Nurs Stud. 2012;49:1568–81. 10.1016/j.ijnurstu.2012.04.001. 22542267 10.1016/j.ijnurstu.2012.04.001

[56] Ziebolz D, Werner C, Schmalz G, Nitschke I, Haak R, Mausberg RF, et al. Oral Health and nutritional status in nursing home residents-results of an explorative cross-Sectional pilot study. BMC Geriatr. 2017;17:39. 28143415 10.1186/s12877-017-0429-0 PMC5282867

[57] Rauen MS, Moreira EA, Calvo MC, Lobo AS. Oral condition and its relationship to nutritional status in the institutionalized elderly population. J Am Diet Assoc. 2006;106:1112–4. 10.1016/j.jada.2006.04.015. 16815129 10.1016/j.jada.2006.04.015

[58] Sheiham A, Steele JG, Marcenes W, et al. The Relationship among Dental Status, Nutrient Intake, and Nutritional Status in Older People. J Dent Res. 2001;80:408–13. 10.1177/00220345010800020201. 11332523 10.1177/00220345010800020201

[59] Dion N, Cotart JL, Rabilloud M. Correction of nutrition test errors for more accurate quantification of the link between dental health and malnutrition. Nutrition. 2007;23:301–7. 10.1016/j.nut.2007.01.009. 17360158 10.1016/j.nut.2007.01.009

[60] Office for National Statistics. Social Survey Division, Information Centre for Health, and Social Care, 2012, Adult Dental Health Survey, 2009, [data collection], UK Data Service, 2nd Edition, Accessed 11 June 2018. SN: 6884, 10.5255/UKDA-SN-6884-2.

[61] Public Health England, (2015b). What is Known About the Oral Health of Older People in England and Wales A review of oral health surveys of older people. Oral health of older people in England and Wales.GOV.UK. (www.gov.uk).

[62] Office for Health Improvement and Disparities. Adult Oral Health Survey, 2021. Self -reported health of teeth and gums. Published 2024. https://www.gov.uk/government/statistics/adult-oral-health-survey-2021/adult-oral-health-survey-2021-self-reported-health-of-teeth-and-gums.

[63] National Institute for Health and Care Excellence. Oral Health for adults in Care Homes. NICE Guideline NG48. Published 2016. Overview | Oral health for adults in care homes | Guidance | NICE.

[64] Langley J, Wassall R, Geddis-Regan A, Watson S, Verey A, McKenna G, et al (2022). Putting guidelines into practice: Using co‐design to develop a complex intervention based on NG48 to enable care staff to provide daily oral care to older people living in care homes. Gerodontology. 40. 10.1111/ger.12629. 10.1111/ger.12629 35288971

[65] Scottish Government. An Action Plan for Improving Oral Health and Modernising NHS Dental Services in Scotland. Published 2005. An Action Plan for Improving Oral Health and Modernising NHS Dental Services in Scotland - gov.scot (www.gov.scot).

[66] Edwards, M (2017). NHS Scotland’s Caring for Smiles: improving the oral health of adults in care settings in line with NICE Guidance and Quality Standards. In NSL Database (Ed). https://www.nice.org.uk/sharedlearning/nhs-scotland-s-caring-for-smiles-improving-the-oral-health-of-adults-in-care-settings-in-line-with-nice-guidance-and-quality-standards.

[67] Welsh Government. Improving Oral Health for Older People Living in Care Homes in Wales: Gwên am Byth programme. Gwên.am.byth.Public.Health.Wales.(nhs.wales). Accessed March 2024.

[68] Care Quality Commission. Smiling Matters: Oral health in care homes. (2019). Smiling matters: oral health care in care homes - Care Quality Commission.(cqc.org.uk).

[69] Department of Health and Social Care. Faster, simpler and fairer: our plan to recover and reform NHS dentistry. (2024). https://www.gov.uk/government/publications/our-plan-to-recover-and-reform-nhs-dentistry.

[70] Al-Ansari A. Is professionally applied fluoride effective in preventing or arresting caries in older adults? Evid Based Dent. 2022;23:138–9. 10.1038/s41432-022-0841-y. 36526834 10.1038/s41432-022-0841-y

[71] Mitchell C, Gross AJ, Milgrom P, Mancl L, Prince DB. Silver diamine fluoride treatment of active root caries lesions in older adults: A case series. J Dent. 2021;105:103561. 10.1016/j.jdent.2020.103561. 33347946 10.1016/j.jdent.2020.103561

[72] Subbiah GK, Gopinathan NM. Is Silver Diamine Fluoride Effective in Preventing and Arresting Caries in Elderly Adults? A Systematic Review. J Int Soc Prev Community Dent. 2018;8:191–9. 10.4103/jispcd.JISPCD_99_18. 29911054 10.4103/jispcd.JISPCD_99_18 PMC5985673

[73] Monaghan N, Morgan M. Oral health policy and access to dentistry in care homes. J Disabil Oral Health. 2010;11:61–68.

[74] Guidelines for the oral healthcare of older people living in nursing and residential homes in Northern Ireland. In. (GAIN). (2012). https://www.rqia.org.uk/RQIA/files/12/12a65998-23a4-4610-a1f6-afe6ce2fa059.pdf.

[75] National Institute of Clinical Excellence (NICE). Dental checks: intervals between oral health reviews: Clinical guideline. Published: 27 October 2004. www.nice.org.uk/guidance/cg19. 31869036

[76] Patel R, Mian M, Robertson C, Pitts NB, Gallagher JE. Crisis in care homes: the dentists don’t come. BDJ Open. 2021;7:20. 10.1038/s41405-021-00075-4. 34103478 10.1038/s41405-021-00075-4 PMC8186358

[77] Kleinman ER, Harper PR, Gallagher JE. Trends in NHS primary dental care for older people in England: implications for the future. Gerodontology. 2009;26:193–201. 10.1111/j.1741-2358.2008.00260.x. 19545327 10.1111/j.1741-2358.2008.00260.x

[78] Allen F, Fan Si, Loke W, Na T, Yan G, Mittal R. The relationship between self-efficacy and oral health status of older adults. J Dent. 2022;122:104085. 10.1016/j.jdent.2022.104085. 35248673 10.1016/j.jdent.2022.104085

[79] Borreani, E, Wright, D, Scambler, S, & Gallagher, JE (2008). Minimising barriers to dental care in older people. BMC Oral Health, 8, 7. 10.1186/1472-6831-8-7. 10.1186/1472-6831-8-7 PMC2335092 18366785

[80] Johnson IG, Morgan MZ, Monaghan NP, Karki AJ. Does dental disease presence equate to treatment need among care home residents? J Dent. 2014;42:929–37. 10.1016/j.jdent.2014.05.010. 24887362 10.1016/j.jdent.2014.05.010

[81] Holmes RD, Burford B, Vance G. Development and retention of the dental workforce: findings from a regional workforce survey and symposium in England. BMC Health Serv Res. 2020;20:255. 10.1186/s12913-020-4980-6. 32216779 10.1186/s12913-020-4980-6 PMC7099783

[82] General Detnal Council (UK). Dentists’ working patterns data as part of the Dentist Annual Renewal (2023). https://www.gdc-uk.org/about-us/our-organisation/reports/working-patterns-data.

